# Can elearning be used to teach palliative care? – medical students’ acceptance, knowledge, and self-estimation of competence in palliative care after elearning

**DOI:** 10.1186/s12909-018-1186-2

**Published:** 2018-04-27

**Authors:** Christian Schulz-Quach, Ursula Wenzel-Meyburg, Katharina Fetz

**Affiliations:** 1 Institute of Psychiatry, Psychology and Neuroscience (IoPPN), Department of Psychological Medicine, King’s College, London, UK; 2 Interdisciplinary Centre for Palliative Medicine, Medical Faculty, Heinrich Heine University Dusseldorf, Dusseldorf, Germany; 3 St Christopher’s Hospice London, Dusseldorf, UK; 4 Chair of Research Methodology and Statistics in Psychology, Department of Psychology & Psychotherapy, Faculty of Health, Witten/Herdecke University, Witten, Germany

**Keywords:** Medical education, Undergraduate palliative care education, Palliative care, Palliative medicine, Education assessment, Evaluation

## Abstract

**Background:**

Undergraduate palliative care education (UPCE) was mandatorily incorporated in medical education in Germany in 2009. Implementation of the new cross-sectional examination subject of palliative care (QB13) continues to be a major challenge for medical schools. It is clear that there is a need among students for more UPCE. On the other hand, there is a lack of teaching resources and patient availabilities for the practical lessons. Digital media and elearning might be one solution to this problem. The primary objective of this study is to evaluate the elearning course *Palliative Care Basics*, with regard to students’ acceptance of this teaching method and their performance in the written examination on the topic of palliative care. In addition, students’ self-estimation in competence in palliative care was assessed.

**Methods:**

To investigate students’ acceptance of the elearning course *Palliative Care Basics*, we conducted a cross-sectional study that is appropriate for proof-of-concept evaluation. The sample consisted of three cohorts of medical students of Heinrich Heine University Dusseldorf (*N* = 670). The acceptance of the elearning approach was investigated by means of the standard evaluation of Heinrich Heine University. The effect of elearning on students’ self-estimation in palliative care competencies was measured by means of the German revised version of the Program in Palliative Care Education and Practice Questionnaire (PCEP-GR).

**Results:**

The elearning course *Palliative Care Basics* was well-received by medical students. The data yielded no significant effects of the elearning course on students’ self-estimation in palliative care competencies. There was a trend of the elearning course having a positive effect on the mark in written exam.

**Conclusions:**

Elearning is a promising approach in UPCE and well-accepted by medical students. It may be able to increase students’ knowledge in palliative care. However, it is likely that there are other approaches needed to change students’ self-estimation in palliative care competencies. It seems plausible that experience-based learning and encounters with dying patients and their relatives are required to increases students’ self-estimation in palliative care competencies.

**Trial registration:**

Heinrich Heine University Medical School Clinical Trial Registry No. 4876 (date of approval 26.11.2014).

## Background

Undergraduate palliative care education (UPCE) was mandatorily incorporated in medical education in Germany in 2009 [1]. Implementation of the new cross-sectional examination subject of palliative care (QB13) continues to be a major challenge for medical schools [2]-The discrepancy between student numbers, limited teaching resources, and clinically and ethically acceptable patient contact is also a challenging task in the development of palliative care teaching [3]. However, numerous studies describe students’ explicitly and clearly communicated suggestions. Students reported only limited confidence regarding their knowledge base in palliative care, and felt rather non-confident or non-confident in communicating the change from a curative treatment to palliative care to patients, or the treatment and provision of care for terminally ill patients [4]. Students asked for well-grounded basic teaching and more lessons on end-of-life care and role modelling from residents and attendings [5, 6]. Students wished for more opportunities and time to deal with their own emotions and to adequately meet patients’ needs and wishes [7]. Rhodes-Kropf found that medical students experienced patient deaths as emotionally powerful even when they were not close to the patients and they felt inadequately supported by their supervisors [8]. Students also expressed the wish to be provided with a self-awareness training and the opportunity to reflect on their own death [9].

In light of the above, there is a need among students for more UPCE. On the other hand, there is a lack of teaching resources and patient availability for the practical lessons. Digital media and elearning might be one solution to this dilemma. The use of electronic devices in medical education has been widely accepted [10, 11]. Consequently, students evaluate internet-based learning as useful [12, 13]. According to our knowledge, the use of elearning in UPCE has not yet been fully investigated. It has been investigated in first studies [14, 15] but never in Germany and never on a large scale. Hence, we developed a UPCE elearning course comprising ten teaching units.

The primary objective of this study is to evaluate the elearning course *Palliative Care Basics* with regard to students’ acceptance of this teaching method and their performance in the written examination on the topic of palliative care. In addition, students’ competence in palliative care was assessed by means of PCEP-GR [16]. It was hypothesised that the acceptance of this new approach would be high. Regarding the effect of elearning on students’ self-estimation and knowledge concerning palliative care, we had four major hypotheses:

1. Elearning increases students’ self-estimation of competence in communication with dying patients and their relatives.
2. Elearning increases students’ self-estimation of knowledge and skills in palliative care.
3. Elearning increases students’ preparation to provide palliative care.
4. Elearning increases students’ knowledge in palliative care (measured by the mark in the written examination).

## Methods

### Data collection

Data was collected either after participation in the elearning course or after individual preparation by means of textbooks and self-guided learning and prior to the written examination on the topic of palliative care. All students who participated in the multiple choice examination were previously informed about the study via email. Students were informed via written and oral communication that their participation in the study was on a voluntary basis. The response rate of the questionnaires was 97%. Twenty students chose not to participate in this study.

### Sample

The study sample consisted of three cohorts. In the first study cohort, 329 (49.1%) undergraduate medical students completed the elearning course during the summer semester of 2013. The second study cohort consisted of 222 (33.1%) students completing the course during the winter semester of 2013/2014, and 119 (17.8%) students completed the elearning-course in the summer semester of 2014, resulting in a total of 670 participants for all three cohorts. The students’ average age was 28 years. The participants’ average duration of studies was 11 semesters. The participants were 65.5% (*n* = 439) female and 34.5% (*n* = 231) male.

### Study design

To investigate students’ acceptance of the elearning course, we conducted a cross-sectional study that is appropriate for proof-of-concept evaluation. Data was collected via written self-assessment. Ethical approval was obtained from the Ethics Committee of the Medical Faculty of Heinrich Heine University Dusseldorf, Germany (Ethics committee approval No. 4876). The study was conducted in accordance with the Declaration of Helsinki on Ethical Principles for Medical Research involving Human Subjects. This report complies with the Strengthening the Reporting of Observational Studies in Epidemiology (STROBE) 22-item checklist to improve the quality of reporting of observational research [17].

### Independent variables

#### *Elearning course:* Palliative care basics

The elearning course consists of five contextual teaching domains: symptom management, communication and interaction, interprofessionalism, ethical/legal/societal aspects, and self-reflection. Thematic contents and didactic methods of the elearning course are presented in Table 1. In addition to the content of these modules, the course includes multiple-choice questions to help students prepare for the final exam. The included reflection questions are aimed at providing the participating students with the opportunity to reflect on their own attitudes towards death and dying.

**Table 1:** Thematic content and didactic methods of the elearning course Basic Topics in Palliative Care

Module	Contents	Teaching units (TU)	Didactic methods
Introduction	Fundamentals of palliative care Communication and psychosocial aspects	2 TU	Virtual Standardised Patients (VSPs) electures; graphics, Youtube videos Self-reflection exercises
Module 1: Symptom management: pain, dyspnea	WHO-staging system, opioid rotation, therapy of dyspnea	1 TU	Virtual Standardised Patients (VSPs) electures
Module 2: Breaking bad news in palliative care	Communication in palliative care (e.g. SPIKES model)	1 TU	Learning from models
Module 3: Nutrition and thirst at the end-of-life	Eating, drinking and oral care	1 TU	Interprofessional teaching
Module 4: Gastroenterological symptom management	Nausea, vomiting, constipation, obstruction, diarrhoea	1 TU	Problem-based learning with case vignettes
Module 5: Psychiatric symptom management	Anxiety, delirium	1 TU	Virtual Standardised Patients (VSPs) electures; Interactive case management
Module 6: Interprofessional Team	Members of the palliative care team	1 TU	Virtual Standardised Patients (VSPs) Interprofessional Teaching
Module 7: Clinical ethics	Advance care planning, aspects of euthanasia	1 TU	Interactive case management
Module 8: Symptom management: final phase	Dying phases, Liverpool Care Pathway	1 TU	Virtual Standardised Patients (VSPs) electures
		Total 10 TU	

1 TU = 45 min

The elearning course comprises ten teaching units (TU) and was developed for clinical medical students. It is part of the mandatory palliative care curriculum and is taught on an interdisciplinary and interprofessional basis to fourth- and fifth-year medical students at Heinrich Heine University Dusseldorf, Germany. All students who had been enrolled in the elearning course were included in the study. The technical realisation of the course was effected via the Casus online platform®. The elearning course is based on systematic education research and the deliberate use of elements promoting successful learning in virtual teaching environments (engendering affective impressions, the experience of success, and quick successions of user repetition) in order to effectively teach highly sensitive and complex issues of palliative care [18, 19]. In this course, the use of virtual standardised patient contact (VSPC) and interprofessional teaching constitute the central didactic elements [20]. Other educational methods and tools used are e-lectures, patient case vignettes, and reflective study questions with experts’ answers. A multiple choice test at the end of the module prepares students for the mandatory final examination. The participating students completed the paper-based questionnaires immediately before the exam.

### Dependent variables

#### PCEP questionnaire

The effect of elearning on students’ self-estimation in palliative care was measured by means of the German revised version of the *Program in Palliative Care Education and Practice Questionnaire* [PCEP-GR, 16]. PCEP-GR was developed on the basis of the *Program in Palliative Care Education and Practice Questionnaire* of Harvard Medical school [21].

Items were translated into German and the questionnaire was shortened to a set of 36 items measured on a 5-point Likert-scale It includes four subscales: *preparation to provide palliative care*, *attitudes towards palliative care*, *self-estimation of competence in communication with dying patients and their relatives,* and *self-estimation of knowledge and skills in palliative care*. Additionally, it provides a PCEP-GR global index for the global evaluation of UPCE programs. A recent validation study [16] investigating PCEP-GR questionnaires’ psychometric properties revealed a good content validity, a good split-half-reliability of the global index (Spearman-Brown-Coefficient = .90), and acceptable reliability indices of the subscales (Cronbach’s alpha = .66–.83).*Acceptance questionnaire.*

Students’ acceptance of the elearning course was determined by means of the obligatory course evaluation of Heinrich Heine University. Standard items in this questionnaire have been developed by the medical faculty and have not yet been validated for their psychometric properties. The questionnaire consists of ten items focussing on the preparation for the exam (*elearning, textbook, other preparation,* and *none*), evaluation of didactic elements, and an overall grading. Items were scored on a five-point Likert-scale (range: 0–5). An additional free-text field provided students with the opportunity to express their own wishes and suggestions regarding the elearning course.

#### Written examination

Finally, a 20-item multiple choice questionnaire (multiple choice test) was used for the purpose of assessment and verification of the acquired knowledge.

#### Statistical analysis

All statistical analyses were performed using IBM SPSS 22. Data was controlled for missing values and plausibility prior to analyses. Two students were excluded from the sample due to missing values. The PCEP global index and subscales were calculated following the suggestion of [16]. The descriptive statistics are reported. Prior to inferential analyses, data was controlled for normal distribution by means of the Kolmogorov-Smirnov Test (KS-Test), and homogeneity of variances by means of Levene’s Test. The KS-Test revealed significant deviation from the normal distribution (all *p*-values < .00). Therefore, hypotheses 1–4 were tested by means of nonparametric Mann-Whitney-U-Tests with elearning *(yes* vs *no*) as independent variables, and self-estimation of competence in communication with dying patients and their relatives, self-estimation of knowledge and skills in palliative care, and preparation to provide palliative care and knowledge (mark in the written exam) as dependent variables, respectively. Because of multiple comparisons, the statistical level of significance was Bonferroni-adjusted to *p* < .01.

## Results

### Descriptives

592 (99.5%) students made a statement about their elearning behaviour. 569 (96.1%) used elearning as preparation for the exam. 23 (3.4%) did not use elearning for exam preparation. Descriptive statistics for elearners and non-elearners concerning students’ self-estimation of competence in communication with dying patients and their relatives, self-estimation of knowledge and skills in palliative care, preparation to provide palliative care, and knowledge in palliative care are shown in Table 2.

**Table 2:** Mean PCEP-GR subscale scores and PCEP-GR index scores for elearners and non-elearners

Scale	Non-Elearner	Elearner	Overall
Preparation to provide palliative care	3.29 (0.54)	3.36 (0.53)	3.37 (0.54)
Self-estimation of competence in communication with dying patients and their relatives	3.18 (0.56)	3.12 (0.62)	3.14 (0.62)
Self-estimation of knowledge and skills in palliative care	3.38 (0.70)	3.54 (0.56)	3.53 (0.59)
Knowledge (mark in the exam)	1.83 (0.85)	1.80 (0.81)	1.83 (0.85)

Mean (SD)

### Acceptance questionnaire

Acceptance was measured on a Likert scale between 1 (= high acceptance) to 5 (=low acceptance). Students’ acceptance of the elearning course in UPCE was high (*n* = 680; mean = 1.8 [1–5]; SD = 0.8). In preparation for the examination, 83,8% (*n* = 570) of the students completed the elearning course and 7.4% (*n* = 45) used the textbook *Basics in palliative care*. 35,3% (*n* = 240) of the students used other teaching materials to prepare for the examination and 1.3% (*n* = 9) did not prepare for the examination. Upon completion of the elearning course, the students felt well-prepared for the examination (n = 570; mean = 3.9 [1–5]; SD = 0.8); and the included multiple choice questions were regarded as useful to prepare for the written examination (*n* = 563; mean = 4.2 [1–5]; SD = 0.8).

The elearning format made it easier for the students to approach the difficult topics of palliative care (*n* = 578; mean = 4.3 [1–5]; SD = 0.8), they reported increased interest in palliative care issues (*n* = 577; mean = 3.9 [1–5]; SD = 1), and they reported the elearning course as being fun (*n* = 559; mean = 3.9 [1–5]; SD = 0.9). The inclusion of case studies was regarded as helpful (*n* = 579; mean = 4.3 [1–5]; SD = 0.8). The students also reported other elements of the course as being helpful, i.e. the inclusion of sequences of the video “I see you” (*n* = 568; mean = 3.9 [1–5]; SD = 1.1), the electure sequences (*n* = 566; mean = 3.8 [1–5], SD = 1.1), and other included video sequences (e.g. Youtube videos) (*n* = 569; mean = 3.7 [1–5]; SD = 1.1).

### Inferential statistics

The Mann-Whitney-U-Tests yielded no significant results (all *p*-values > .01) supporting hypothesis 1–4. Students who prepared for the exam by means of elearning (M = 1.8) had better marks in the exam than students who did not prepare by means of elearning (M = 2.5); however, this effect did not reach statistical significance (U = − 2.34; *p* = .02). Test statistics of the Mann-Whitney U Tests and corresponding p-values are presented in Table 3.

**Table 3:** Test Statistics of Mann Whitney U-Tests

dependent variable	Mann-Whitney U	*p*
Preparation to provide palliative care	−0.79	.43
Self-estimation of competence in communication with dying patients and their relatives	−0.42	.68
Self-estimation of knowledge and skills in palliative care	−1.17	.24
Knowledge (mark in the exam)	−2.34	.02

## Discussion

There are still discrepancies between existing teaching resources and the high number of students in UPCE. Hence, a new model of elearning teaching delivery in palliative care was introduced and tested. In Germany, most curricula today only describe overall learning objectives, while a specific curriculum and a detailed teaching objective catalogue are absent [22]. There is also a discrepancy between desire and reality with respect to the number of teachers and students [23]. Against this background, the innovative elearning concept “teaching palliative care using virtual standardised patients (VSP)” was developed as part of a curricular development cycle for UPCE at the University of Dusseldorf [24].

The aim of the current study was to investigate medical students’ acceptance of the elearning approach to *Palliative Care Basics*. Our results suggest that elearning is well-accepted by medical students. Another aim of this study was to evaluate the effects of elearning on students’ self-estimation of competence in communication with dying patients and their relatives, self-estimation of knowledge and skills in palliative care, preparation to provide palliative care, and knowledge (measured by the mark in the written exam). The data did not yield significant results for our hypothesis that elearning would increase these factors. We were not able to detect any effects of elearning on students’ self-estimation of competence in communication with dying patients and their relatives, self-estimation of knowledge and skills in palliative care, or preparation to provide palliative care. This may be due to the fact that these factors are more practical aspects of the construct of competence in palliative care. It is possible that elearning is not the appropriate approach to gain practical competency, and that real life contact with dying patients and their relatives is necessary to gain such skills, and consequently to increase self-estimation of competence in communication with dying patients and their relatives, self-estimation of knowledge and skills in palliative care, and preparation to provide palliative care.

We were able to show that elearning is a practical way to teach palliative care topics. The elearning course was well-received by students and has the potential to increase knowledge in palliative care. Other studies have shown similar effects [3, 24]. Successful elearning formats and methods necessitate adequate and reliable technical support and a suitable structural environment. It is of critical importance that elearning teaching units may be accessed and completed via various mobile devices. Such new technologies create more mobility, while on the other hand necessitating a higher level of technical prerequisites, which may quickly lead to user frustration without adequate support [3].

The results of the present study are limited on the basis of the methodological restrictions of a proof-of-concept study. The unique survey does not allow causal assumptions but is suited to exploring new concepts. Acceptance of the presented concept was high. However, the results also show the limitations of online teaching in palliative care education. Students ask for instruction and training regarding attitudes toward death and dying through experience-based teaching [25]. The students’ free-text responses revealed that students accepted and appreciated the elearning course, but that the elearning contents could not replace direct encounters and practice, which is why the students expressed their wishes for a higher degree of real patient contact. These results correlate with other studies [26]. Gibbins showed that students’ suggested including real patients in UPCE.

Blended learning models might be used as a middle course between students’ expectations and wishes, and structural limitations. According to a study on student evaluations of a blended course, [15] demonstrated the success of a blended-learning approach in UPCE in comparison to a traditional face-to-face teaching approach. Ruiz et al. highlighted evidence for the effectiveness and acceptance of elearning within medical education, in particular with regard to the blended-learning approach combining elearning and traditional face-to-face teaching formats [10]. Blended learning formats also enable educators to realistically design and edit online tools about topics and situations which do not lend themselves to traditional teaching formats (e.g. final phase, rituals following the death of a loved one, and family conflicts) [27]. In light of the increasing use of elearning in medical education, further studies are deemed necessary and therefore highly recommended.

### Limitations

We admit that questionnaire-based evaluation of the effects of elearning courses in UPCE has its limitations. The self-efficacy expectation and knowledge of the construct of competence in palliative care include practical aspects in providing palliative care and communication with patients and relatives. Consequently, it would be helpful to supplement questionnaire-based evaluation of elearning courses in UPCE programs by means of objective clinical examinations [28, 29] in order to gain valuable behavioural outcome data [30–33]. Due to limited resources this option is often not feasible at university medical centres.

The study also has some methodological limitations. There were no baseline measurements of students’ knowledge and perceived self efficacy in palliative care prior to the elearning course. Consequently, no pre- vs post-elearning analyses concerning the dependent variables were possible. Due to missing sample characteristics no subgroup analyses concerning age and gender were possible. Due to inhomogeneous sample sizes of subgroups (elearners vs. non-elearners) results need to be interpretated with caution. It is possible that we were not able to detect an effect due to a type two error (is the failure to reject a false null hypothesis).

## Abbreviations

OSCE: objective structured clinical examination
PCEP-GR: German revised version of the Program in Palliative Care Education and Practice Questionnaire
UPCE: undergraduate palliative care education
